# Determination of the Size of the Roots of Teeth Previous to Extraction

**Published:** 1869-09

**Authors:** 


### Determination of the Size of the Roots of Teeth previous to Extraction.

Before the educated dentist attempts the extraction of a tooth, he examines the form of the crown, which enables him to determine with certainty the direction of the roots. For young practitioners and students, some indications will be of importance, therefore I give here, those communicated by Dr. B. Whener:

I.—If the crown is large and short we may expect that the roots are long, while with a long and narrow crown, the roots are small and slender.

II.—If the neck of the posterior tooth is much thinner than its crown, the roots will diverge.

III.—If the neck of a posterior tooth is as large as its crown, we may conclude that the roots run down parallel with the sides of the crown.

IV.—In case the neck of a posterior tooth should be larger than the grinding surfaces, the roots will be found converging.

V.—When we observe one of the sides of the crown inclining to the middle of the tooth, so we will find the corresponding root bent in the same direction, while the other roots are found parallel with the perpendicular line of the tooth.

In the wisdom teeth the abnormal direction of the roots is the most common.—*Am. Jour. Dental Science.—Canada Jour. Dental Science.*

